# Comparative polar and lipid plasma metabolomics differentiate KSHV infection and disease states

**DOI:** 10.1186/s40170-023-00316-0

**Published:** 2023-08-31

**Authors:** Sara R. Privatt, Camila Pereira Braga, Alicia Johnson, Salum J. Lidenge, Luke Berry, John R. Ngowi, Owen Ngalamika, Andrew G. Chapple, Julius Mwaiselage, Charles Wood, John T. West, Jiri Adamec

**Affiliations:** 1 School of Biological Sciences, University of Nebraska-Lincoln, Lincoln, NE USA; 2 Department of Interdisciplinary Oncology, Louisiana State University Health Sciences Center, New Orleans, LA USA; 3 Department of Biochemistry, University of Nebraska-Lincoln, Lincoln, NE USA; 4 Redox Biology Center, University of Nebraska-Lincoln, Lincoln, NE USA; 5 Ocean Road Cancer Institute, Dar Es Salaam, Tanzania; 6 Muhimbili University of Health and Allied Sciences, Dar Es Salaam, Tanzania; 7 Dermatology and Venereology Section, Adult Hospital of the University Teaching Hospitals, University of Zambia School of Medicine, Lusaka, Zambia

**Keywords:** Kaposi sarcoma, KSHV, HIV, Metabolite profiling, Biomarkers, Metabolomics

## Abstract

**Background:**

Kaposi sarcoma (KS) is a neoplastic disease etiologically associated with infection by the Kaposi sarcoma-associated herpesvirus (KSHV). KS manifests primarily as cutaneous lesions in individuals due to either age (classical KS), HIV infection (epidemic KS), or tissue rejection preventatives in transplantation (iatrogenic KS) but can also occur in individuals, predominantly in sub-Saharan Africa (SSA), lacking any obvious immune suppression (endemic KS). The high endemicity of KSHV and human immunodeficiency virus-1 (HIV) co-infection in Africa results in KS being one of the top 5 cancers there. As with most viral cancers, infection with KSHV alone is insufficient to induce tumorigenesis. Indeed, KSHV infection of primary human endothelial cell cultures, even at high levels, is rarely associated with long-term culture, transformation, or growth deregulation, yet infection in vivo is sustained for life. Investigations of immune mediators that distinguish KSHV infection, KSHV/HIV co-infection, and symptomatic KS disease have yet to reveal consistent correlates of protection against or progression to KS. In addition to viral infection, it is plausible that pathogenesis also requires an immunological and metabolic environment permissive to the abnormal endothelial cell growth evident in KS tumors. In this study, we explored whether plasma metabolomes could differentiate asymptomatic KSHV-infected individuals with or without HIV co-infection and symptomatic KS from each other.

**Methods:**

To investigate how metabolic changes may correlate with co-infections and tumorigenesis, plasma samples derived from KSHV seropositive sub-Saharan African subjects in three groups, (A) asymptomatic (lacking neoplastic disease) with KSHV infection only, (B) asymptomatic co-infected with KSHV and HIV, and (C) symptomatic with clinically diagnosed KS, were subjected to analysis of lipid and polar metabolite profiles

**Results:**

Polar and nonpolar plasma metabolic differentials were evident in both comparisons. Integration of the metabolic findings with our previously reported KS transcriptomics data suggests dysregulation of amino acid/urea cycle and purine metabolic pathways, in concert with viral infection in KS disease progression.

**Conclusions:**

This study is, to our knowledge, the first to report human plasma metabolic differentials between in vivo KSHV infection and co-infection with HIV, as well as differentials between co-infection and epidemic KS.

**Supplementary Information:**

The online version contains supplementary material available at 10.1186/s40170-023-00316-0.

## Background

Kaposi sarcoma (KS) is a neoplastic disease first characterized in elderly Mediterranean men by the Hungarian physician Moritz Kaposi [1–3]. The disease occurs endemically at high incidence in sub-Saharan Africa as well as parts of the Mediterranean Basin and in specific regions of South America [4–6]. In association with the acquired immune deficiency syndrome (AIDS) pandemic, KS came to be widely recognized as an AIDS-defining condition. In fact, prior to the isolation of Kaposi sarcoma-associated herpesvirus (KSHV) or human herpesvirus-8 (HHV-8), it was the abnormally high incidence of this rare sarcoma among men who have sex with men (MSM) that triggered the search for the infectious agent that ultimately became the human immunodeficiency virus-1 (HIV) in the 1980s [7]. We now know that KS requires infection with the gammaherpesvirus KSHV [1]. There are four forms of KS, all of which are attributable to KSHV infection, growth dysregulation, and abnormal angiogenesis in endothelial cells. This includes classic KS (cKS), as originally observed in elderly Mediterranean men, endemic KS (EnKS)—in men, women, and children lacking HIV co-infection; iatrogenic KS (iKS)—resulting from chemical immunosuppression in organ transplantation; and epidemic KS (EpKS)—resulting from HIV co-infection [4, 8, 9]. In addition, KSHV infection is also linked to B-cell neoplastic diseases such as primary effusion lymphoma (PEL), multicentric Castleman’s disease (MCD), and KSHV-induced inflammatory cytokine syndrome (KICS) [4].

Although KSHV seroprevalence is low in the USA and Europe, it is significantly higher in specific populations such as MSM and those co-infected with HIV [10]. Antiretroviral therapies (ART) have been highly successful in the USA and Europe in reducing the incidence of EpKS, consistent with a role for immune dysfunction in KS development. Treatment responses vary depending on age, staging, visceral involvement, co-infections and comorbidities, and other sociodemographic variables [11, 12]. Unfortunately, KS presentation is often advanced in sub-Saharan Africa, despite HIV viral suppression. The response rate of such advanced patients to treatment with chemotherapy is ~ 50% in Zambia, of whom an additional 50% succumb to recurrence within a year. Currently, there are no prognostic markers or tests that can provide any indication of likely treatment success or failure, other than the HIV disease staging parameters and the AIDS clinical trials group (ACTG) initiated tumor/immune/system (T/I/S) staging criteria often being used. Moreover, no markers are reliable indicators of the transition from asymptomatic to symptomatic presentation or of protection against such progression.

Recent transcriptomics studies comparing tumor biopsy gene expression to that of normal skin from the same individuals have revealed several salient KS features [13, 14]. First, HIV transcripts are rarely detected in KS tumor tissue, suggesting HIV gene products are not direct drivers of tumorigenesis. Second, EpKS and EnKS upregulate or downregulate a majority of the same genes and do so in the same direction, differing only in the greater magnitude of that dysregulation in EnKS. This finding implies that HIV lowers the threshold for malignant transformation. Moreover, gene dysregulation in EpKS is shared between subjects with undetectable HIV plasma viral load and those with active HIV replication. Whether such dysregulation of expression is communicated into detectable plasma metabolite differentials is one of the objectives of the investigation here. Our previous RNA-Seq data revealed significant changes in glucose metabolism, the Krebs (tricarboxylic acid) cycle, and multiple aspects of lipid metabolism. Additionally, the data suggested activation of redox balance to promote cell growth as opposed to virus-induced dysregulation. Increases in angiogenesis pathways and evidence of immune signaling consistent with attempted T-cell recruitment were all associated with KS tumorigenesis [15, 16]. Our follow-up studies have, however, failed to identify a robust immune infiltration of KS tumors, and limited cytokine differences were detectable between EnKS and EpKS with the differentials predominantly in immune-regulatory, suppressive, or Th2-skewing, as opposed to hyper-inflammatory, categories [17, 18].

Previous studies investigating the effects of KSHV infection on cell metabolism have been restricted to in vitro infections over a short time course. Dysregulation of glycolysis, fatty acid synthesis, and glutaminolysis was evident during both latency and lytic reactivation [19–22]. Glycolysis and glutaminolysis were suggested to be essential for KSHV viral replication in vitro, and inhibition of these pathways prevented the generation of new infectious virus [23]. In human tissue, the relationships between virus replication and KS disease progression are less apparent since in most KS tissues, gene expression from the latency locus predominates. Limited cellular transcriptomic overlap (10%) between KS tissues and telomerase-immortalized human microvascular endothelial (TIME) cells, a commonly used in vitro infection model, has been previously reported [13]. Nevertheless, the most predominantly shared pathway between in vitro models and KS tumors was glucose metabolism disorder, reflective of widespread metabolic reprogramming commonly observed in viral infection and cancer.

The lack of data characterizing in vivo KSHV/HIV co-infection-associated and KS-specific metabolic dysregulation underscored a need to assess potential diagnostic and prognostic implications of metabolic dysregulation in KSHV-induced neoplastic disease. In this study, comparisons of polar and nonpolar (lipid) plasma metabolites in KSHV infection versus co-infection with HIV (KSHV + /HIV − vs. KSHV + /HIV +), and between asymptomatic co-infection with symptomatic KS (KSHV + /HIV + vs. EpKS), were conducted to preliminarily determine if differentials metabolites among them existed. This approach defined metabolites unique to co-infection and tumorigenesis that may have potential as marker panels for disease staging and treatment outcome. Moreover, the integration of tumor transcriptomics with the plasma metabolome pointed to specific pathways that become additively or synergistically dysregulated in the pathogenetic transition of KSHV infection to malignant disease with or without the contribution of HIV co-infection. An increased appreciation of these KS pathogenetic pathways may provide a framework for novel combinatorial approaches perhaps with dose-sparing regimens to target the viral, metabolic, oncologic, and immunologic aspects of the KS disease.

## Materials and methods

### Sample collection

Twenty-four participants (> 18 years old) were recruited from the Ocean Road Cancer Institute (ORCI), Tanzania, as described in Lidenge et al. [14]. Participants (both genders) were 18 years and older. Whole blood was collected in EDTA tubes followed by plasma isolation by centrifugation at 400 × *g* for 5 min. Plasma was stored at − 80 °C until shipment. HIV viral load and CD4 quantification were performed as described in Lidenge et al. [17]. This study was approved by the review boards of the Tanzania National Institute for Medical Research, Ocean Road Cancer Institute, the University of Nebraska Medical Center, the University of Nebraska-Lincoln, and the Louisiana State University Health Sciences Center.

### Sample extraction

From each individual, 20 µL of plasma was used to generate polar and nonpolar (lipid) metabolite fractions. Each plasma sample was transferred to a 1.5-mL Eppendorf tube, and 500 µL of methanol and 30 µL of ribitol (2 mg/mL in water, used as an internal standard) were added as a spike control. Samples were vigorously mixed, incubated for 15 min at 70 °C, and centrifuged at 14,000 × *g* for 10 min. The supernatant (500 µL) was transferred to a new tube with 250 µL of chloroform and 500 µL of water and mixed vigorously for 1 min. To separate polar and nonpolar phases, the samples were centrifuged at 1500 × *g* for 15 min, and each phase was transferred to separate tubes. Extracted polar metabolites (upper aqueous phase, 500 µL) were dried in a speed vacuum before derivatization and GC–MS analysis. Extracted nonpolar lipid metabolites (lower chloroform phase, 200 µL) were dried under a constant nitrogen stream and dissolved in 50 µL of methanol just before LC–MS global lipid profiling analysis. In addition to the samples, blanks and quality control samples were prepared for both LC and GC–MS platforms. A blank sample was prepared without the addition of plasma but underwent all the extraction steps for the elimination of potential contaminants coming from non-biological background sources (solvents, tubes, etc.) during the sample processing and preparation. Quality control (QC) samples were prepared by mixing equal aliquots of each sample and injected between biological samples to evaluate sensitivity, stability, and reproducibility of the LC and GC–MS acquisition platforms. Samples were analyzed in random order.

### Lipid profiling

Lipid analyses were carried out using an Agilent 1200 Series high-performance liquid chromatography (HPLC) system coupled to a high-resolution/accuracy Fourier-transform ion cyclotron resonance (FT-ICR) 7.05 T mass spectrometer (MS) (Bruker Daltonics, Germany). An aliquot of 5 µL of the methanol resuspended, dried chloroform extraction was injected onto an ACE 5 C8-300 column (2.1 × 100 mm) column, and lipids were separated in a linear gradient elution at a flow rate of 0.1 mL/min. The mobile phases consisted of A: 0.1% formic acid and 10-mM ammonium acetate in Milli-Q water and B: 0.1% formic acid and 10-mM ammonium acetate in acetonitrile/ isopropanol (50/50; v/v). The gradient was set as follows: *T* = 0 min: 30% B; *T* = 1 min: 30% B; *T* = 25 min: 100% B; *T* = 45 min: 100% B; *T* = 47 min: 30% B; and *T* = 60 min: 30% B (column re-equilibration). Data was collected in positive mode in a scan range of 244–1800 m/z and 0.2 s of ion accumulation time with estimated resolving power of 78,000 (at m/z 400). A capillary voltage of 4500 V, and end-plate offset of − 500 V, was used. Dry temperature and gas flow were set at 180 °C and 4 L/min, respectively. CompassXport v. 3.0.6 (Bruker Daltonics, Germany) software was used to convert LC–MS data to a general mzXML format and further processed with the mzMine software package for peak detection, deconvolution, normalization, and alignment [24].

### Profiling of polar metabolites

For polar analysis, dried samples were derivatized by addition of 40-μL methoxyamine hydrochloride (20 mg/mL in pyridine), incubated in a shaker for 2 h at 37 °C, followed by the addition of 70-µL N-trimethylsilyl-N-methyl trifluoroacetamide (MSTFA, 1 mL, and 20-µL fatty acid methyl esters (FAME)) and incubation in a shaker for an additional 30 min at 37 °C. After cooling to room temperature, the samples were centrifuged at 14,000 × *g*, for 10 min, and 90 μL of supernatant was transferred into a 200-μL conical base inert glass insert inside a 2-mL amber autosampler glass vial (Agilent Technologies, Germany). MS acquisitions were done using 7200 GC-QTOF system (Agilent) and an HP-5MS UI column (30 m, 0.25 mm, and 0.25 mm; Agilent). The temperature gradient included a starting temperature of 80 °C held for 2 min, increasing at a rate of 15 °C per minute to 350 °C followed by a final hold for 6 min (total run time of 26 min). The ion source temperature was set to 250 °C, while the scanning mass range was set to 60–600 m/z. Acquired data were converted to.abf using AbfConverter and further processed by MS-Dial 4.12 [25].

### Data analysis

Following data processing, Excel files (containing *m/z* values, retention times, and peak areas information for each feature) were generated and exported from MZmine and MS-DIAL. Missing values were imputed using K-nearest neighbors (KNN) and auto-scaled prior to data analyses. Multivariate and univariate statistical analyses were performed using MetaboAnalyst software v5.0 [26, 27]. Using the auto-scaled data from MetaboAnalyst, heatmaps were generated using the R package Pheatmap v1.0.12 in RStudio v2022.02.3 with R v4.2.0.

Principal component analysis (PCA) and Kruskal-Wallace analysis were used to visualize the variation of datasets between groups (GraphPad Prism v9). Polar and nonpolar data were combined, and comparisons between two groups were performed, and volcano plots were constructed. In addition, Wilcoxon ranked-sum analysis with and without a Bonferroni correction was applied. Polar features were tentatively identified using the public Fiehn database search on MS-DIAL before statistical analysis [28]. Nonpolar features with a significant threshold of *p* < 0.05 and a fold-change (FC) threshold of 1.5 (log2 FC of 0.585 < or > 0.585) were tentatively identified on the Human Metabolome Database (HMDB) with a mass tolerance of 10 ppm selecting M + H^+^, M + NH_4_^+^ as the positive ion adducts for nonpolar metabolites [29]. KEGG IDs were obtained for polar and nonpolar tentatively identified metabolites and used to check pathways affected based on the conditions analyzed, using *Homo sapiens* as the organism. In cases where multiple ions could be annotated to the same feature (due to different adducts or ppm), they were counted additively to the predicted pathway classification where at least 2 metabolites were used for the enrichment analysis.

### Pathway analyses integrating metabolomics and transcriptomics data

Pathway analyses were completed in MetaboAnalyst v5.0 using the Joint Pathway Analysis tool [30]. Briefly, significantly changed genes and metabolites were entered without their corresponding fold changes. Gene symbols that did not have a match were removed. The parameters chosen were degree centrality and integrated metabolic pathways (regulatory pathways were not considered). Transcriptomic data used for pathway analyses in this report were previously published by Lidenge et al. [14]. Of this data, genes that possessed a significant threshold of *p* < 0.05 (raw *p*-value) and a fold change (FC) > 2 for KS/control (or lesion/normal skin) or an FC > 1.5 for KS origin (EnKS vs. EpKS) were included. For metabolomics data, identified metabolites with a minimum FC of 2 (*FC* > 2 or < 0.5) and a raw *p*-value < 0.05 were used for pathway analyses. Pathways that were not relevant to humans were excluded in reporting final results (i.e., porphyrin and chlorophyll which is common for plants).

For the KSHV + /HIV + vs. EpKS pairwise comparison, metabolomic data was integrated with comparative transcriptomic data that subtracted normal skin gene expression from that of the KS lesion from 24 subjects used. The integration was conducted to seek insight into KS progression/development biomarkers and how these changes might be reflected in the more accessible plasma.

## Results

### Overall analysis and data overview

**Fig. 1:**
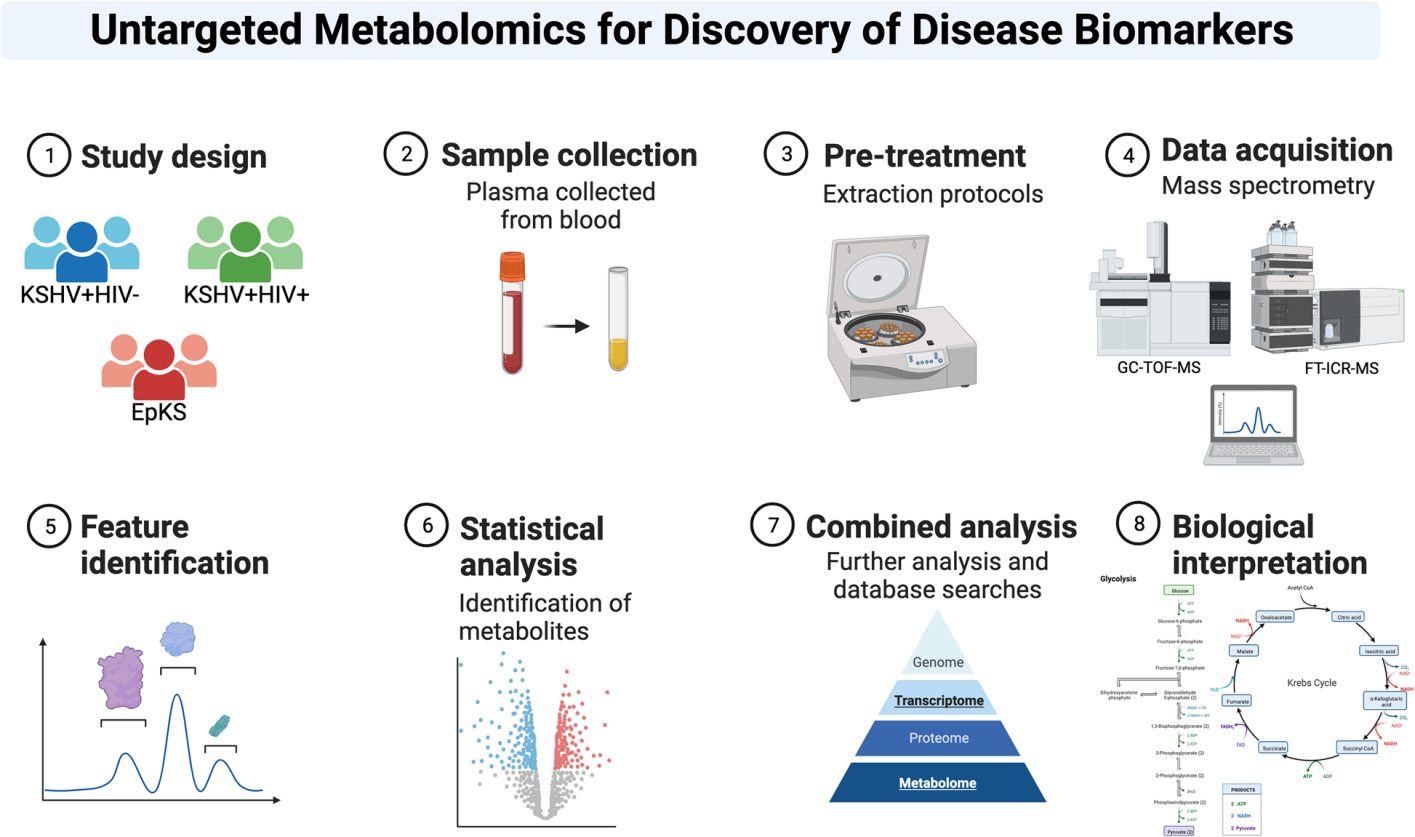
Overview of project design. The 22 individuals were divided into ASY KSHV + HIV − (*N* = 6), ASY KSHV + HIV + (*N* = 7), and SYM EpKS (*N* = 9) groups. Metabolites were extracted from plasma, acquired by GC-TOF–MS and FT-ICR-MS, and then analyzed for biological relevance

Twenty-four KSHV samples were included in the study (Table 1). Of those, 11 samples were KS symptomatic (9 *ep*idemic and 2 *en*demic), and 13 samples were asymptomatic and segregated into groups based on KSHV and HIV serostatus: 7 KSHV^+^/HIV^+^ and 6 KSHV^+^/HIV^−^. In addition, 5 US-collected KSHV^−^/HIV^−^ serum samples were used as controls to monitor variations in technical processing and for quality assurance purposes but were not included in group/pairwise analyses since they are unlikely to be appropriate comparators for parameters (diet, lifestyle, etc.) beyond KSHV or HIV infection status factors. Quality control (QC) samples, representing a pool of all samples analyzed, were used to evaluate technical variation and chromatographic shifts during both LC–MS and GC–MS analyses. The overview of the project workflow is summarized in Fig. 1. Following processing, normalization, and scaling, MetaboAnalyst and Prism 9 were used to produce PCA plots, volcano plots, and other statistical analyses.

**Table 1:** Cohort characteristics. There were no significant differences between group ages or CD4 counts within the HIV + patients

Group	Study ID	Age	Gender	HIV VL/mL	CD4 count
***EnKS***	*21,147*	*39*	*Male*	*NA*	*NA*
***EnKS***	*21,182*	*57*	*Male*	*NA*	*NA*
EpKS	21,117	38	Female	3450	130
EpKS	21,119	39	Male	ND	264
EpKS	21,120	56	Male	14,500	482
EpKS	21,121	42	Male	6310	30
EpKS	21,145	26	Male	NR	422
EpKS	21,161	38	Male	148,000	710
EpKS	21,204	58	Male	ND	261
EpKS	21,220	30	Female	NR	67
EpKS	21,228	38	Female	NR	333
KSHV + HIV −	21,525	48	Male	NA	256
KSHV + HIV −	21,526	50	Male	NA	310
KSHV + HIV −	21,527	54	Male	NA	931
KSHV + HIV −	21,528	55	Male	NA	303
KSHV + HIV −	21,559	33	Female	NA	1054
KSHV + HIV −	21,562	33	Female	NA	1398
KSHV + HIV +	21,570	36	Female	NR	500
KSHV + HIV +	21,575	34	Male	NR	675
KSHV + HIV +	21,578	38	Male	NR	316
KSHV + HIV +	21,579	43	Male	NR	240
KSHV + HIV +	21,580	48	Female	NR	295
KSHV + HIV +	21,582	34	Female	NR	594
KSHV + HIV +	21,583	35	Female	NR	124

*NA* not applicable, *ND* not detectable, *NR* no record

**Fig. 2:**
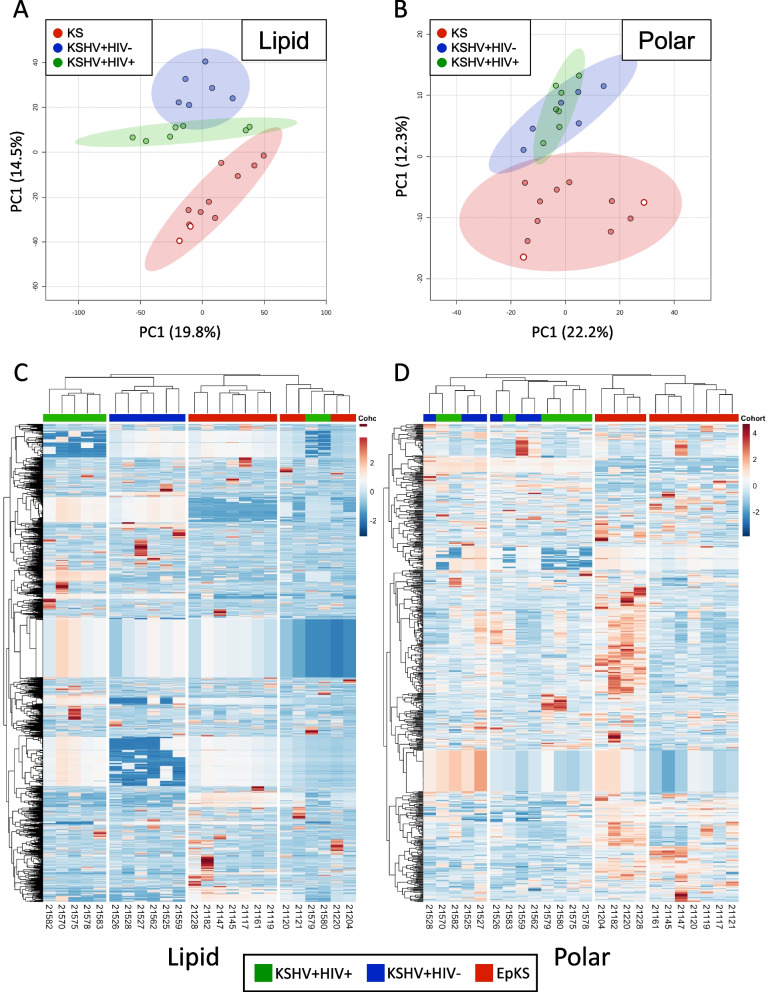
Plasma samples from KS patients are different from KSHV-infected asymptomatic controls. **A** PCA analysis of the lipid profiles and **B** polar metabolites, from all three groups, reveal segregation of the KS subjects’ metabolites. **C** Hierarchal clustering and heatmaps of all detected lipids and **D** all detected polar metabolites using Pearson coefficient and complete clustering. Red, KS cohort; blue, KSHV + HIV − cohort; green, KSHV + HIV + cohort. Endemic KS cases (HIV negative) are indicated in the PCA plots by open red circles

An initial analysis evaluating technical variation showed well-defined clusters of QC samples for both LC- and GC–MS platforms and indicated good reproducibility and reliability of generated data (Supplemental Fig. 1A and B, respectively). Further analyses were therefore carried out without QC samples. PCA plots of lipid and polar metabolites revealed separation between the KS group and asymptomatic groups (Fig. 2A and B). Further clustering analysis showed a well-defined separation between the KS and the asymptomatic individuals in both the lipid and polar metabolites (Fig. 2C and D). Interestingly, two KSHV + /HIV + samples (21,579 and 21,580) clustered with SYM samples in lipid analysis. This may reflect the transition from asymptomatic to symptomatic presentation. Consistent with previous transcriptomic profiling, the two EnKS cases clustered within the EpKS profiles suggesting the plasma metabolite profile is driven primarily by the symptomatic KS disease presentation as opposed to HIV infection status. However, the small sample number prevented us from conducting statistically valid pairwise comparisons between EnKS and EpKS samples. Despite their overall similarity, to avoid potential confusion, the EnKS metabolite profiles have been excluded from analyses hereafter, and we will only present EpKS analysis.

### Analysis of differential polar profiles

**Fig. 3:**
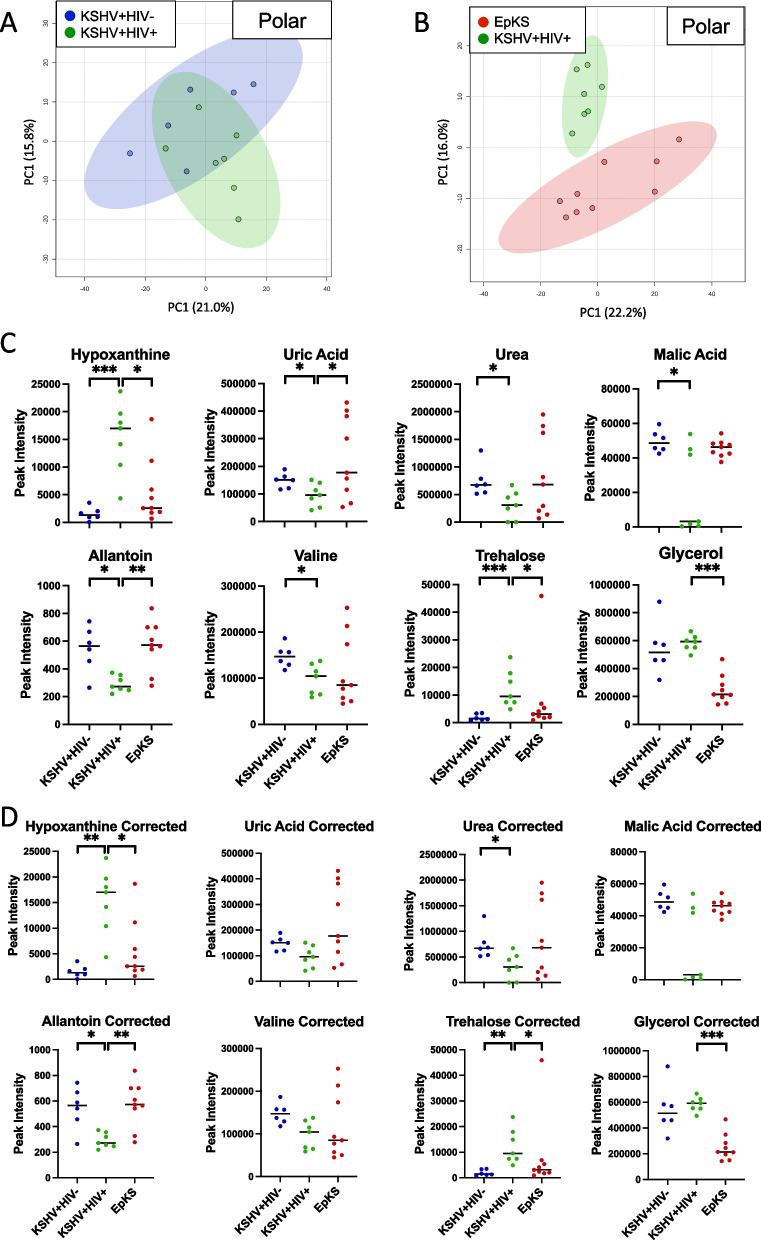
Pairwise comparisons reveal differential features driving segregation. **A** Separation of polar metabolite profiles between KSHV + HIV − and KSHV + HIV + cohorts. **B** Polar metabolite profiles of EpKS and KSHV + HIV + cohorts separate completely. **C** Wilcoxon rank-sum tests of 8 selected differential metabolites without Bonferroni corrections. **D** Wilcoxon rank-sum tests of 8 selected differential metabolites with Bonferroni corrections. Upon correction, uric acid and valine were no longer significantly different despite showing significance in the uncorrected analysis. Red, KS cohort; blue, KSHV + HIV − cohort; green, KSHV + HIV + cohort. *P < 0.05, **P < 0.01, ***P < 0.001

In asymptomatic KSHV-infected individuals, factors such as HIV co-infection increase the risk of progressing to malignancy (EpKS). Multivariate analysis of KSHV^+^HIV^+^ vs KSHV^+^HIV^−^ showed partial segregation between groups for polar features (Fig. 3A) suggesting a relatively minor impact of HIV + coinfection on changes in overall profiles. Wilcoxon rank-sum tests, however, revealed 55 significantly changed polar features underlying the segregation between the KSHV^+^HIV^+^ and KSHV^+^HIV^−^ groups (*p*-value < 0.05) with a fold change of − 0.585 < or > 0.585. Of the 55 identified features, only 12 could be confirmed using the Fiehn database for GC–MS. Of the 12 discriminant features, 4 metabolites were upregulated in the context of HIV co-infection, and 8 were decreased (Supplemental Table 1).

To investigate whether plasma metabolite differentials were evident between symptomatic EpKS and asymptomatic KSHV^+^HIV^+^ co-infection, an unsupervised PCA analysis was performed (Fig. 3B). The resulting separate clusters indicate significant changes in polar metabolic profiles based on the transition from co-infection to symptomatic disease. This segregation was confirmed by pairwise analysis (Wilcoxon rank-sum tests) that revealed 89 significantly different polar features. These included 14 metabolites that were identifiable and confirmed using the Fiehn database, 9 of which were upregulated in EpKS and 5 were downregulated. The most differential identified metabolite was glycerol which is central in both carbohydrate and lipid metabolism (Supplemental Table 1).

Combined results from both comparisons revealed 20 significantly differential and identifiable polar metabolites, 7 of which were attributable to the presence/absence of HIV co-infection (the KSHV^+^HIV^+^ vs KSHV^+^HIV^−^ comparison) and another 8 that were differential based on the presence or absence of neoplastic disease (the EpKS vs KSHV^+^HIV^+^ comparison). Five differential polar metabolites were common to both comparisons suggesting interconnectivity between co-infection-associated metabolic changes and pathways involved in KSHV tumorigenesis. Distribution and trends of the most relevant metabolites for both groups are shown in Fig. 3C indicating an important role of purine metabolism in both ASY KSHV infection and KS tumorigenesis.

### Analysis of differential lipid profiles

**Fig. 4:**
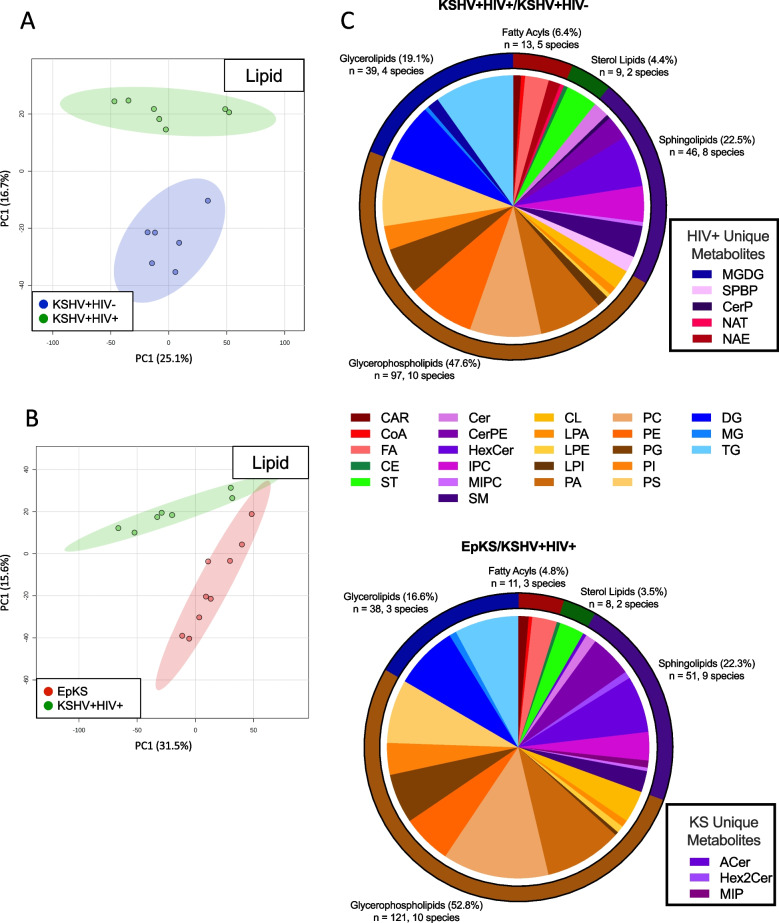
A pairwise comparison of lipid profiles highlights both common and distinct differential lipid families. PCA plots of lipid metabolite profiles between **A** KSHV + HIV − and KSHV + HIV + cohorts and **B** EpKS and KSHV + HIV + cohorts showing complete separation. Red, KS cohort; blue, KSHV + HIV − cohort; green, KSHV + HIV + cohort. **C** Breakdown of differential lipid metabolites from both pairwise comparisons. Each wedge represents a lipid class. Comparison unique metabolites are highlighted in the boxes to the right

Multivariate analysis of lipid metabolites showed well-defined clustering in both KSHV^+^HIV^−^ *vs*. KSHV^+^HIV^+^ and KSHV^+^HIV^+^ *vs*. EpKS comparisons (Fig. 4A and B, respectively). Because the signal was collected in MS-only mode, no structural details are available, and potential metabolite ID(s) can only be reported by atomic composition without specific details of side chain length, double bond position, or similar. Tentative identification of individual features was done by matching measured m/z with the predicted molecular mass of metabolites in the Human Metabolome Database (HMDB). For some features, multiple IDs corresponded, and therefore, these features are designated as molecular classes rather than specific compounds. Using this approach, a total of 204 and 229 differential lipids between KSHV + HIV + and KSHV + HIV − , and between EpKS and KSHV + HIV + individuals, respectively, were tentatively identified/classified. These represent 32 different lipid classes as summarized in Fig. 4C. Although some of these classes are plant specific, they are listed as they can reflect dietary and/or lipid uptake specifics or potentially reflect the known impact of HIV on gut integrity and microbial translocation that presumably also applies to metabolite translocation.

Because of the seminal roles sphingolipids and glycerophospholipids play in viral structure, replication cycles, viral curvature, viral assembly, and viral entry into target cells, the dynamics of their metabolites are of particular interest in KSHV infection and disease. While the number of identified sphingolipids was not different between comparisons, monogalactosyldiacylglycerols (MGDG), sphingoid base-phosphates (SPBP), and ceramide-phosphates (CerP) were differential in the asymptomatic comparison, but acyl ceramides (ACer), di-hexosylceramides (Hex2Cer), and mannosyl-phosphoramides (MIP) replaced these as differential families in the KS vs. KSHV + HIV + comparison. The proportion of dysregulated glycerophospholipids increased in neoplasia but not in response to HIV co-infection. Interestingly, one of the highly differential molecules for both comparisons is cardiolipin, indicating a potential impact on mitochondrial membranes and their integrity in either immune cells or potentially affected tissue [31].

### Correlation between identified metabolites and KS transcriptomics

**Fig. 5:**
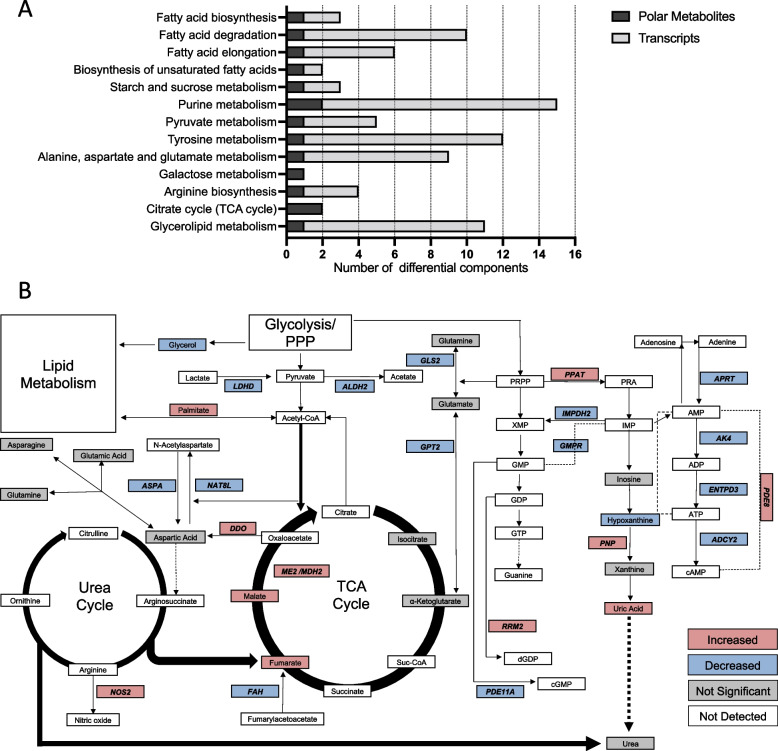
Pathway analysis integrating expression differentials from KS transcriptomics with differential polar metabolites between KS and KSHV + HIV + individuals. For visualization purposes, the authors have focused on pathways in which both genes and metabolites were identified for the purpose of linking metabolic and gene expression phenotypes. **A** Pathway analyses were completed using the MetaboAnalyst Joint Pathway Analysis tool in MetaboAnalyst v5.0 [27]. For metabolomics data, identified metabolites with a minimum FC of 2 (*FC* > 2 or < 0.5) and a raw *p*-value < 0.05 were used for pathway analyses. Included transcriptomics data was derived from a previously published report in which the transcriptomic profile of healthy skin was compared to cutaneous KS lesions [14]. **B** A summary of significantly changed genes and metabolites within central carbon, purine, and urea metabolism is depicted. Genes and metabolites related to lipid metabolism have not been included due to redundancy and space limits. Red- and blue-colored boxes indicate increased and decreased detection, respectively, while gray boxes indicate metabolites that were detected in this study but were not significantly different. Genes are bold and italicized

In previously published work, both glycolysis and lipid metabolism pathways were highly dysregulated at the transcriptomic level [13]. We next integrated our transcriptomic and metabolomic data to evaluate the extent to which dysregulated gene expression in KS tumor tissue was being reflected in plasma metabolite profiles and to gain preliminary insight into the potentially dysregulated pathways. For this analysis, the differential polar metabolites from the EpKS vs. KSHV + HIV + comparison were analyzed in conjunction with all cellular transcripts evincing > twofold differential expression between KS tumor and normal uninvolved skin in comparisons from 24 individuals (*N* = 1104 transcripts). KEGG pathway analysis revealed the most correlative, dysregulated pathways to be amino acid synthesis, purine, and pyruvate metabolism (Fig. 5A). A more detailed analysis revealed a combination of up-and downregulated metabolites and genes (Fig. 5B), particularly in the metabolism of uric acid, which may be explained by the downregulation of purine nucleoside phosphorylase (*PNP)*, the enzyme which breaks down hypoxanthine.

## Discussion

In this study, plasma provides a partial, albeit dilute, conduit of information that reflects to some extent the pathogenesis-associated metabolic profiles of KSHV-affected cells and tissue(s). Overall, distinct metabolic profiles from each of the three experimental groups were identified with a clear separation between symptomatic and asymptomatic individuals. Most of the differential features were identified from the lipid (nonpolar) fraction suggesting significant effects in lipid metabolism, a finding consistent with the highly dysregulated pathways highlighted from transcriptomics of KS tumors [13]. Although the lipidomics method employed here could not reveal the exact structures of these featured molecules, the molecular mass of precursor ions and retention time were used to identify their atomic composition and lipid classes. We are using this preliminary data to reduce the complexity of future KSHV/KS lipid analyses that would also include tumor tissue and employ a more targeted MS–MS approach that would support complete characterization. Glycerophospholipids and sphingolipids represented more than 75% of the differential lipids (Fig. 5). In cancer, lipid metabolism plays a crucial role in membrane rearrangement, energy production, and signaling molecules, and cancer cells are well known for their increased uptake, scavenging, and de novo synthesis leading to the presence of unusual and/or exogenous lipids [32]. This is reflected by the identification of 229 differential lipids in KS samples indicating increased uptake and de novo synthesis. However, transcriptomics suggested a profound downregulation in most lipid pathways including anabolic, catabolic, and lipid storage domains. Thus, the metabolomic data may imply more of a lipid scavenging role of the KS tumor regarding lipid utilization, especially since the tumor cells are not robustly replicating KSHV and therefore are not likely producing lipids in an effort to support viral assembly components. Additionally, MIP was detected in KS samples which can be indicative of dysregulated signaling pathways such as the phosphoinositide 3-kinase-protein kinase B (PI3K-AKT) pathway and can directly affect the activity of lipogenic enzymes and thus the lipid composition, particularly phosphatidylinositol (PI) [33, 34]. Increased lipogenesis can be also suggested by decreased levels of glycerol which together with glycerol 3-P represents an essential precursor for glycerophospholipids biogenesis. In fact, Villumsen et al. found differential diglyceride (DG) and triglyceride (TG) metabolites associated with metabolism disorders within HIV-infected individuals, supporting our findings that HIV infection and subsequent tumorigenesis both alter the plasma lipidome [35].

The polar fraction was analyzed by GC–MS, allowing in some cases to support the confirmed identification of altered metabolic features. Many changes were related to energy/redox balance and purine metabolism both of which converge to support low-level viral replication and cancerous growth due to metabolic reprogramming [36]. Within the purine metabolism pathway, hypoxanthine and uric acid were significantly affected by cancer development where hypoxanthine was decreased, and uric acid was significantly increased in the EpKS group. This correlates with the previous finding of 13 dysregulated genes from those pathways identified by transcriptomic analysis (Fig. 5). Other studies have also identified decreased levels of hypoxanthine as being indicative of cancer progression in colorectal cancer and lung cancer [37, 38].

Reprogramming of energy metabolism and redox balance are hallmarks of tumorigenesis in general [39]. High demand for ATP and increased levels of reactive oxygen species (ROS), together with hypoxic conditions in tumor tissue, lead to the Warburg effect, a balancing act involving glycolysis generating ATP and the pentose phosphate pathway (PPP) maintaining reducing power together with supplying precursors for purine metabolism [36]. Cancer cells exhibit higher levels of ROS production and consequently increased levels of ROS scavenging [40]. Trehalose, detected as differentially increased in KSHV + /HIV + versus KSHV + HIV − plasma, is well known for its antioxidant properties with anti-inflammatory and cancer-inhibitory effects [41]. In addition, it targets and inhibits the mTOR pathway, thereby inducing autophagy and reducing viral entry in human primary macrophages and CD4 + T cells [41]. The increase of trehalose in KSHV + /HIV + and a slight increase in EpKS samples compared to KSHV + /HIV − samples may reflect an attempt to limit HIV infection, but since trehalose cannot be synthesized by human metabolism, its levels must reflect an HIV-associated availability which may be associated with loss of gut integrity [42]. In EpKS, the trehalose levels could be decreased by its utilization in tumor energy metabolism and attempted redox balance.

The Warburg effect as induced by viral infections and cancer can also induce metabolic reprogramming that manifests in changes to TCA cycle flux. The respective increases of plasma malate, fumarate (Fig. 5), and the gene for malate dehydrogenase 2 (*MDH2*) in EpKS are suggestive of increased anaplerotic flux into the TCA cycle. KSHV-infected endothelial cells were shown to have increased glutamine uptake which is required for glutaminolysis as it feeds α-ketoglutarate to the TCA cycle which promotes alternative energy production in cancer cells [21, 43]. This appeared to accompany transcriptomic changes in glutamine metabolism (Fig. 5), which has been suggested by others in KSHV in vitro infections [23]. Because the Warburg effect results in the preferential production of lactate, the TCA cycle must derive its intermediates from alternative sources such as amino acids. Interestingly, lactate is inhibitory to T-cell infiltration and function, which is consistent with the poor infiltration of CD8 + T cells that we have previously reported in KS tissues [44]. However, as a function of chemoattractant chemokines by those same tissues, CD8 T cells are found on KS tumor margins [16], so it will be important in future studies using KS tissue to investigate the levels of lactate accumulation in correlation with lymphocyte infiltrations.

Purine metabolism is commonly dysregulated in viral infection and cancer as both conditions require increased nucleotide availability to sustain growth, replication, and survival [45]. Indeed, nucleotide metabolism has long been an antiviral and antineoplastic therapeutic target [45]. However, this shared characteristic may have different metabolic underpinnings depending on latency, the presence of coinfections, and if/when KSHV progresses to KS. The opposing patterns observed in the mono-infection, coinfection, and KS progression comparisons may be attributed to competition for metabolic resources between infected cells, cancer cells, infected transformed cells, and normal cells. Chang et al. reported a metabolic competition for glucose between T cells and cancer cells in a mouse model of sarcoma that aided cancer progression [46]. Similar patterns may be evident in the opposing hypoxanthine and uric acid patterns from the KSHV + /HIV − vs. KSHV + /HIV + and EpKS vs. KSHV + /HIV + comparisons. This is also supported by changes in allantoin, an oxidation product (non-enzymatic in humans) of uric acid, where we observed low levels in KSHV + /HIV + and equal levels in KSHV + HIV − and EpKS. In the KSHV + /HIV − vs. KSHV + /HIV + comparison, the increase in hypoxanthine and decrease in uric acid may indicate increased purine salvage. This was demonstrated by Vastag et al., who showed that pyrimidine metabolites peaked around 12-h post HSV1 infection followed by a decrease [47]. Elevated serum uric acid has been correlated with increased inflammation in cohort studies [48, 49]. In KSHV + /HIV + vs. EpKS comparison, the increase in *PNP* in tumors and uric acid in plasma may indicate increased inflammation, promoting tumorigenesis [50]. Alternatively, the ability of uric acid to act as an antioxidant may serve to protect cancer cells, as it is well established that the increased metabolic requirements of tumor microenvironments produce higher levels of ROS [50]. This increased metabolic rate is also suggested by the elevation in several TCA cycle genes and metabolites.

The patients’ diet likely played a large role in which metabolites were detectable. During the statistical analysis, we identified citramalic acid as being increased in EpKS compared to the KSHV + /HIV + group. Citramalic acid is a metabolite that does not typically appear in human metabolic studies; however, it is associated with eating or drinking fruit-based products or alcoholic beverages, as it is part of fermentation. Additionally, a study investigating the effects of chemotherapy on the plasma metabolome in breast cancer patients before and after chemotherapy found a loose association with lipid dysregulation and increased detection of citramalic acid [51]. This supports what we have seen in KS where there is an abundance of lipid dysregulation observed in both the tumor transcriptome and the plasma metabolome. The difference in diets also posed a problem related to controls for this study, as we had initially included uninfected blood donors, albeit from the USA. Initial statistical analysis of this group compared to the KSHV + /HIV − group presented with over 100 differential metabolites, a result that indicated that our groups were far too different to be justifiably compared. Further studies with a larger cohort will be needed to investigate the importance of such rare metabolites and the overall impact of KSHV infection alone.

One final important aspect of KSHV/KS metabolic reprogramming is using amino acids as a source of energy and nitrogen. Of these, decreased valine was evident in KSHV + /HIV + and even more in EpKS. Valine is a branched-chain amino acid (BCAA) that can be converted into acetyl-CoA and further metabolized in the TCA cycle or diverted as a precursor in lipogenesis. It is also a major nitrogen source for glutamine synthesis. As an essential amino acid, levels of valine reflect protein digestion, degradation, and uptake of amino acids. Decreasing levels in EpKS suggest increased cellular biogenesis and energy needs in neoplasia. Interestingly, another product of protein degradation, urea, exhibited decreased levels in KSHV + /HIV + but returned to the KSHV + /HIV − levels in EpKS. This might be explained by reprogramming to maximize the utilization of nitrogen for anabolic macromolecule synthesis in tumorigenesis.

## Conclusions

Overall, our data speak to discernable plasma metabolic differentials between KSHV infection and co-infection with HIV, as well as to plasma metabolites that mark the progression from co-infection to neoplastic growth of cutaneous KS tumors. These differentials reside in both polar and nonpolar metabolites and show discrete linkages to transcriptomic dysregulation, likely as a function of dilution, as might be expected from plasma sampling tissue metabolites from the entirety of the human body. Unfortunately, our small sample sizes and the unavailability of normal healthy controls from Tanzania made it difficult to control for some confounding factors such as diet, lifestyle, or additional co-infections; however, we were able to demonstrate that there are changes in the overall metabolic profiles of KSHV-infected ASY and SYM individuals. Future studies will attempt to separate more generalized neoplastic markers (those present in any cancer or any cancer with infectious etiology) from markers that may be specific for KS disease progression. Plasma lipid metabolites and their role as potential biomarkers will need further refinement in coupled tissue and plasma analyses now that we have identified rationally targeted pathways and features. Similarly, we hope to be able to focus future investigations on more targeted polar metabolite biomarkers using the refined focus generated here, and coupled tumor, plasma, and perhaps other bodily fluid specimens.

### Supplementary Information

**Additional file 1: Table S1.** Differentially identified metabolites for the two pair-wise comparisons. Analysis was performed using Wilcoxon Rank-Sum tests.

**Additional file 2: Supplemental Fig. 1.** PCA plots demonstrating QC sample separation from symptomatic and asymptomatic individuals.

## Data Availability

All relevant data are included in the manuscript or supplemental materials. Any additional requests can be directed to the corresponding author.

## Abbreviations

KS: Kaposi sarcoma
KSHV: Kaposi sarcoma-associated herpesvirus
SSA: Sub-Saharan Africa
HIV: Human immunodeficiency virus-1
AIDS: Acquired immune deficiency syndrome
HHV8: Human herpesvirus-8
MSM: Men who have sex with men
cKS: Classical Kaposi sarcoma
EnKS: Endemic Kaposi sarcoma
EpKS: Epidemic Kaposi sarcoma
iKS: Iatrogenic Kaposi sarcoma
PEL: Primary effusion lymphoma
MCD: Multicentric Castleman’s disease
KICS: KSHV-induced inflammatory cytokine syndrome
ART: Antiretroviral therapy
ACTG: AIDS clinical trials group
T/I/S: Tumor/immune/system
TIME: Telomerase immortalized microvascular endothelial cells
QC: Quality control
MS: Mass spectrometer
HPLC: High-performance liquid chromatography
FT-ICR: Fourier-transform ion cyclotron resonance
FAME: Fatty acid methyl esters
KNN: K-nearest neighbors
PCA: Principal component analysis
FC: Fold change
HMDB: Human metabolome database
ASY: Asymptomatic
SYM: Symptomatic
MGDG: Monogalactosyldiacylglycerols
SPBP: Sphingoid base phosphates
CerP: Ceramide phosphates
ACer: Acyl ceramides
Hex2Cer: Dihexosylceramides
MIP: Mannosyl phosphoramides
PNP: Purine nucleoside phosphorylase
PI3K-AKT: Phosphoinositide 3-kinase-protein kinase B
PI: Phosphatidylinositol
DG: Diglyceride
TG: Triglyceride
PPP: Pentose phosphate pathway
MDH2: Malate dehydrogenase 2
BCAA: Branched-chain amino acid
ROS: Reactive oxygen species
