# Supplementary material for: Comparative polar and lipid plasma metabolomics differentiate KSHV infection and disease states
Source: Cancer Metab. 2023 Aug 31;11:13. doi: 10.1186/s40170-023-00316-0 (PMC10470137; doi:10.1186/s40170-023-00316-0)
Supplement: Supplementary file 1 — Additional file 1: Table S1. Differentially identified metabolites for the two pair-wise comparisons. Analysis was performed using Wilcoxon Rank-Sum tests. [file 40170_2023_316_MOESM1_ESM.docx]

| **Metabolite Name** | **KSHV+HIV+/KSHV+HIV-** | **EpKS/KSHV+HIV+** |
| --- | --- | --- |
|  | ***p-value*** | ***p-value*** |
| Serine | 0.001 | - |
| Decanoic acid | 0.014 | - |
| Urea | 0.014 | - |
| Valine | 0.022 | - |
| 4-Hydroxyphenylacetic acid | 0.035 | - |
| Bicine | 0.035 | - |
| Malic acid | 0.035 | - |
| Glycerol | - | 0.001 |
| Dehydroabietic acid | - | 0.001 |
| Benzoic acid | - | 0.012 |
| Citramalic acid | - | 0.012 |
| Palmitic acid | - | 0.012 |
| Fumaric acid | - | 0.023 |
| Heptadecanoic acid | - | 0.031 |
| Cerotinic acid | - | 0.042 |
| Hypoxanthine | 0.001 | 0.016 |
| Trehalose | 0.001 | 0.016 |
| Allantoin | 0.014 | 0.003 |
| Pyrophosphate Meox2 | 0.022 | 0.023 |
| Uric acid | 0.022 | 0.042 |

Supplemental Table 1. Differentially identified metabolites for the two pair-wise comparisons. Analysis was performed using Wilcoxon Rank-Sum tests.
