# Supplementary material for: Comparative polar and lipid plasma metabolomics differentiate KSHV infection and disease states
Source: Cancer Metab. 2023 Aug 31;11:13. doi: 10.1186/s40170-023-00316-0 (PMC10470137; doi:10.1186/s40170-023-00316-0)
Supplement: Supplementary file 2 — Additional file 2: Supplemental Fig. 1. PCA plots demonstrating QC sample separation from symptomatic and asymptomatic individuals. [file 40170_2023_316_MOESM2_ESM.docx]

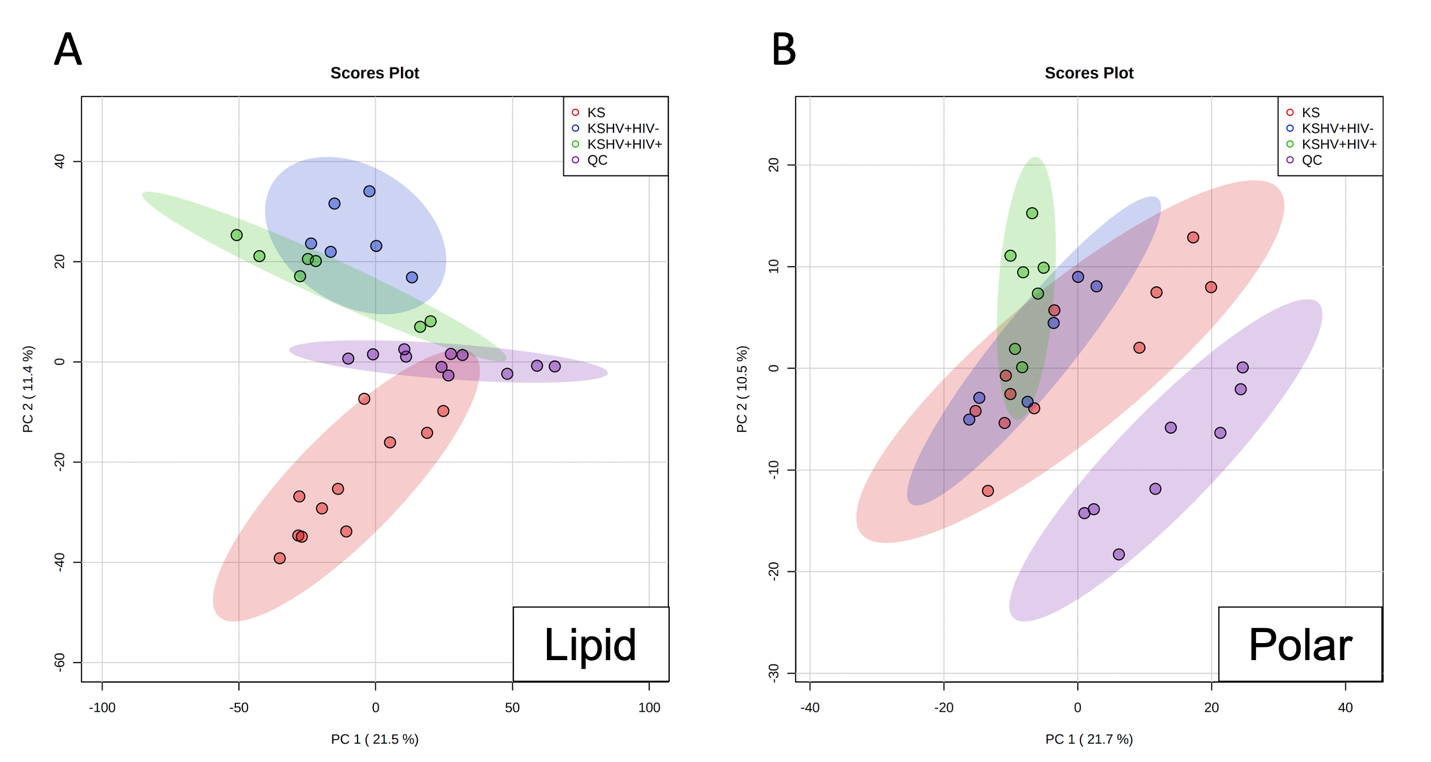


Supplemental Figure 1. PCA plots demonstrating QC sample (purple) separation from symptomatic and asymptomatic individuals.
